# Licorice–wolfberry-derived nanomaterial improves the germination rate of wheat under salt stress by maintaining reactive oxygen species homeostasis

**DOI:** 10.3389/fpls.2025.1657516

**Published:** 2025-09-08

**Authors:** Wenya Wang, Xiaomeng Lian, Zhiqian Li, Linfeng Bao, Jiahao Liu, Tingyong Mao, Desheng Wang, Lili Yang, Long Ma, Lu Han

**Affiliations:** ^1^ College of Agriculture, Tarim University, Alar, China; ^2^ Key Laboratory of Tarim Oasis Agriculture (Tarim University), Ministry of Education, Alar, China; ^3^ Key Laboratory of Saline-alkali Soil Improvement and Utilization (Saline-alkali land in arid and semi-arid regions), Ministry of Agriculture and Rural Affairs, Urumqi, China

**Keywords:** licorice and wolfberry-derived complex nanomaterial, *Triticum aestivum L.*, salt stress, ROS homeostasis, germination

## Abstract

**Introduction:**

The research on improving the salt tolerance of crops through plant nanobiotechnology has been extensively reported. However, the mechanism by which plant - derived nanomaterials enhance the germination rate of wheat under salt stress remains elusive. Unveiling the mechanism by which plant - derived nanomaterials boost the salt tolerance of wheat is conducive to safeguarding food security.

**Methods:**

Herein, we used mesoporous self-assembly licorice and wolfberry-derived complex nanomaterial (LW-CNs) to soak wheat (*Triticum aestivum* L) seeds.

**Results:**

The size and zeta potential of LW-CNs were 42.2±8.2 nm and ^-1^9.6±1.5 eV, respectively. After 4 days of salt stress, LW-CNs-soaked wheat seeds presented a higher germination rate (78.4±8.3 vs 54.4±8.5%) and protein content (44.0±0.1 vs 39.1±0.2 mg g^-1^), but no significant effect was observed on fresh weight (2.6±0.4 vs 2.3±0.4 g). LW-CNs significantly increased the pigment content (*chlorophyll a*: 0.11±0.0 vs 0.03±0.0 mg g^-1^, *chlorophyll b*: 0.05±0.0 vs 0.02±0.0 mg g^-1^, and carotenoids: 10.3±0.0 vs 2.9±0.0 μg g^-1^). LW-CNs alleviated salt-induced reactive oxygen species (ROS) accumulated through increase superoxide dismutase (917.4±8.7 vs 767.5±1.6 U g^-1^), peroxidase (2458.7±5.0 vs 2070.5±14.8 U g^-1^), and catalase (158.3±3.9 vs 112.0±3.2 μmol min^-1^ g^-1^) activity. Soaking in LW-CNs maintained ROS homeostasis also through the ascorbic acid–glutathione cycle. Furthermore, LW-CNs elevated the K⁺/Na⁺ ratio within wheat seeds and augmented the activities of nitrogen metabolism enzymes.

**Conclusion:**

Overall, our study demonstrates that soaking seeds with plant - derived nanomaterials promotes the growth and nutrient absorption of wheat under salt stress by modulating the homeostasis of reactive oxygen species (ROS) and the K⁺/Na⁺ ratio/.

## 1 Introduction

Salt stress significantly impacts agricultural production globally, increasing production costs and reducing yields (Khan et al., 2021). Wheat is an important crop that feeds over 35% of the world’s population and provides carbohydrates (Shewry and Hey, 2015). However, wheat production is affected by salt stress, especially during the germination stage (Nauman Khan et al., 2024). Enhancing the germination rate of wheat under salt stress is a crucial measure for ensuring food security.

The production of reactive oxygen species (ROS) is one of the main factors leading to crop damage under salt stress. Under salt stress, ROS in plant cells may be generated at multiple sites, with the main types being hydrogen peroxide (H_2_O_2_), superoxide anion (O_2_^·—^), hydroxyl radical (·OH), and singlet oxygen (^1^O_2_) (Liu et al., 2021a). The overaccumulation of ROS damages DNA, lipids, and proteins (He et al., 2024; Kruk et al., 2005; Li et al., 2022; Wang et al., 2008). To reduce the damage caused by ROS, plants have developed antioxidant enzyme systems that include peroxidase (POD), catalase (CAT), and superoxide dismutase (SOD), which effectively eliminate H_2_O_2_ and O_2_^·—^ (Alscher, 2002; Laxa et al., 2019). However, ·OH generated by the Fenton reaction between H_2_O_2_ and Fe^2+^ cannot be removed by any known enzymatic system, making it the strongest known oxidizing molecule (Mittler, 2017).

Antioxidant enzymes function on the membrane, and when the membrane is oxidatively damaged, it loses its function. Thus, plants have other metabolic pathways, such as the ascorbate–glutathione cycle (AsA-GSH), to produce antioxidants for compensation (Foyer and Noctor, 2011). In this cycle, AsA and GSH possess strong antioxidant capacities and can be recycled. The study of the AsA-GSH cycle can assist researchers in understanding the role of ROS homeostasis in plants’ salt tolerance (Kaya et al., 2023).

Salinity can lead to nutrient obstruction in crops. Under salt stress conditions, the number of root nodules in soybeans decreases, nitrogen fixation activity is reduced, nutrient absorption is diminished, and growth is inhibited (He et al., 2021). High concentrations of Na^-1^ can cause a decrease in the nitrogenase activity of soybeans (Zilli et al., 2008). This, in turn, hinders the protein synthesis of crops, ultimately affecting the final yield. By foliar spraying of Brassinosteroids, the decline in nitrogenase activity caused by salt stress can be alleviated, thereby ensuring the growth of plants (López-Gómez et al., 2016).As an important metallic nutrient element, potassium (K) serves as the metallic cofactor for more than 60 enzymes within crop cells (Johnson et al., 2022). Under salt stress, the massive influx of Na^-1^ can impede the absorption of K^-1^ and cause its loss, ultimately resulting in cell death. Maintaining a high K^-1^/Na^-1^ ratio is one of the crucial measures to enhance the salt stress tolerance of crops (Liu et al., 2021b).

Plant nanobiotechnology, an interdisciplinary discipline that integrates materials science and botany, presents distinctive concepts for improving the salt tolerance of crops. For instance, researchers have employed cerium oxide, manganese trioxide, and zinc oxide nanoparticles to increase salt tolerance in cotton, rapeseed, cucumber, and rice (Li et al., 2022; Liu et al., 2023, 2021b; Lu et al., 2020; Zhou et al., 2021). Studies showing the enhancement of the germination rate of crops under salt stress through seed treatments mainly employ cerium oxide nanoparticles and selenium-doped carbon dots. However, to the best of our knowledge, there has been no report on the utilization of nano-complexes composed of these two morphologies to enhance the germination rate of crops under salt stress, especially synthesized from herbal. Licorice and wolfberry-derived complex nanomaterial (LW-CNs), a composite material loading wolfberry-derived carbon dots onto licorice-derived mesoporous carbon balls, possesses excellent bactericidal effects and promotes rapeseed growth (Qiu et al., 2025). Furthermore, LW-CNs maintains plant ROS homeostasis under stress and, therefore, might improve plants’ salt tolerance.

This study aimed to elucidate the physiological mechanism by which LW-CNs-treated wheat seeds tolerate salt stress. We hypothesized that LW-CNs not only maintained ROS homeostasis by regulating the antioxidant enzyme system, but also regulated the non-enzymatic antioxidant system, such as the ASA-GSH cycle. This dual regulatory mechanism contributed to an increase in the germination rate of wheat under salt stress.

## 2 Materials and methods

### 2.1 Synthesis and characterization of Li-MSs, Wo-CDs, and LW-CNs

The We synthesized LW-CNs based on our previous study (Qiu et al., 2025). Briefly, 200 mg of licorice or wolfberry powder was added to an autoclave containing 10 mL H_2_O (**Supplementary Figure S1A**), and the mixture was heated at 160°C for 6 h. After heating, the solution was centrifuged at 12,000 rpm for 1 h, and the supernatant was collected, resulting in licorice-based mesoporous spheres (Li-MSs) or wolfberry-based carbon dots (Wo-CDs). For LW-CNs synthesis, 100 mg each of licorice and wolfberry powder was combined in an autoclave with 10 mL of deionized water, and the solution was heated at 160°C for 6 h. The final solution was centrifuged at 12,000 rpm for 1 h, and the supernatant, which contained LW-CNs, was collected. The synthesized nanomaterials were stored at 4°C for future use. High-resolution transmission electron microscopy (HR-TEM) images were captured on a JEM-2100Plus microscope (JEOL Co., Ltd., Japan) with an accelerating voltage of 200 kV. Transmission electron microscopy (TEM) was carried out by adding prepared samples dropwise to a copper mesh. The size and zeta potential of nanoparticles were characterized by a model Nano 90 Malvern zetasizer (Malvern, UK).

### 2.2 ROS-scavenging ability assay of LW-CNs

The O_2_^•—^ scavenge rate was determined following a previously described method (Lu et al., 2020) and calculated using a SOD assay kit (WST-1) (Nanjing Jiancheng Bioengineer, Nanjing, China). First, O_2_^•—^ were produced by xanthine and xanthine oxidase reactions. The generated O_2_^•—^ reacted with WST-1 to make a water-soluble formazan dye, which was detected by measuring the absorbance at 450 nm. LW-CNs was added (final nanoparticle concentration of 200 mg L^-1^) to the mixture of xanthine and xanthine oxidase and then incubated at 37°C for 30 min. The 300 μL suspension was measured at 450 nm using a microplate spectrophotometer (Epoch, Biotek, USA).

The H_2_O_2_ scavenging rate was determined following a previously described method with some modifications (Lu et al., 2020). Briefly, 200 mg L^-1^ LW-CNs was added to H_2_O_2_ (2 mM) solution. After 5 min of incubation at 25°C, the H_2_O_2_ scavenging rate was calculated by monitoring the decrease in H_2_O_2_ absorbance at 240 nm with a microplate spectrophotometer. The molar extinction coefficient was 39.4 M/cm.

### 2.3 Seed materials, seed soaking, stress treatments, and growth conditions

This experiment was completed in the laboratory of the College of Agriculture, Tarim University, Alar (longitude 81.293839° E, latitude 40.539246° N, altitude 1000 m) from March 2024 to February 2025. The wheat (*Triticum aestivum* L.) variety used in this experiment was ‘Xin Dong 55.’ First, 200 mg L^-1^ LW-CNs was used as the soaking medium. Seeds were immersed in LW-CNs, and controls were immersed in water. The Erlenmeyer flask containing the seeds and soaking solution with a seed-to-solution ratio of 1:10 (w/v) was placed on a mechanical shaker (60 rpm) with continuous gentle agitation in the dark for 3 h (To avoid the stress imposed on seeds due to the excessive intake of nanomaterials and in light of the rate of nanomaterials’ entry into seeds during the seed treatment process as described by previous studies, we selected a duration of 3 h). After 3 h, the soaked seeds were sown in a plastic Petri dish (14 cm in diameter, **Supplementary Figure S1B**). Each box contained 40 g of quartz sand and 15 mL of 150 mM NaCl solution. Each box contained 25 seeds and was incubated at 30 ± 1°C during the day and at 25 ± 1°C at night, the relative humidity is 55% and light intensity is 150 μmol·m^-2^·s^-1^ (**Supplementary Figure S1C**). The germination test was terminated 4 days after salt treatment. The number of germinated seeds was recorded daily, and the fresh weight in each box was recorded after 4 days of salt stress, each treatment was recorded with five boxes.

### 2.4 Protein content determination

4 days after salt stress, the protein content of wheat seeds treated with LW-CNs and those untreated was measured separately. Under salt stress conditions, cysteine, cystine, tryptophan, tyrosine, and peptide bonds in proteins can reduce Cu^2+^ to Cu^+^. The Bicinchoninic Acid Assay of the two molecules was combined with Cu^+^ to form a purple complex, which had an absorption peak at 540–595 nm and the strongest absorption peak at 562 nm. The protein content was determined using a protein content assay kit (Mengxi Biomedical Technology Co., Ltd., Jiangsu, China), following the manufacturer’s instructions.

### 2.5 Photosynthetic pigment content determination

After 4 days salt stress, wheat seeds wers used to determined photosynthetic pigment. *Chlorophyll a* and *chlorophyll b* have maximum absorption at 662 and 644 nm, respectively, and the total chlorophyll content was calculated according to the empirical formula. The chlorophyll content in wheat was measured using a plant chlorophyll content determination kit (Mengxi Biomedical Technology Co., Ltd., Jiangsu, China), following the manufacturer’s instructions. The chlorophyll content was obtained using the formula (Liu et al., 2021b).


Chlorophyll a content=9.784×A662−0.99×A644.

1Chlorophyll b content=21.426×A644−4.65×A662

The carotenoid content was determined by the sample extracted by mixed organic solvents. The carotenoids were separated from the non-carotenoid components, and the carotenoids had a maximum absorption peak at 470 nm. The carotenoid content was determined using a kit (Mengxi Biomedical Technology Co., Ltd., Jiangsu, China), following the manufacturer’s instructions.

### 2.6 Determination of the K^+^ content and Na^+^ content

Tissue samples after 4 days of salt stress were deactivated at 105°C for 40 min and dried at 85°C until constant weight.0.1 g of ground dry sample was weighed into a digestion tube, followed by the addition of 5 mL concentrated sulfuric acid for overnight soaking.The samples were heated at 340°C in a digestion furnace for 90 min. When white fumes appeared in the digestion tube, 6-8 drops of 30% H_2_O_2_ solution were added and shaken until colorless, then heated for an additional 60 min to remove excess H_2_O_2_.After digestion, the solution was diluted to 50 mL and filtered through qualitative filter paper. The filtrate was reserved for analysis. Na^-1^ and K^-1^ concentrations were measured using a flame spectrophotometer. The ion content was calculated using the following formula:


$$\text{Ion content }\left({\text{mg g}}^{-1}\text{ FW}\right)=\text{X}*\text{V}/W*1000$$

X represents the absorbance value measured by the flame spectrophotometer; V represents the sample volume during measurement (mL); W represents the dry sample weight weighed (g).

### 2.7 Determination of the GOGAT activity and GS activity

4 days after salt stress, the wheat seeds with and without LW-CNs treatment were sampling to determin the Glutamate synthase (GOGAT) activity and Glutamine synthetase (GS) activity. GOGAT, catalase GS detection kits (Mengxi Biomedical Technology Co., Ltd., Jiangsu, China) were used to determine the GOGAT, and GS activity, respectively. Measurements were made according to the manufacturer’s instructions.

### 2.8 Determination of the ROS content, antioxidant enzyme activity, and malondialdehyde content

4 days after salt stress, the wheat seeds with and without LW-CNs treatment were sampling to determin the ROS content, antioxidant enzyme activity, and MDA content. The H_2_O_2_ and O_2_^.-^ contents in wheat were measured using ROS detection kits. Briefly, the H_2_O_2_ content of wheat seedlings was determined using an H_2_O_2_ assay kit (Mengxi Biomedical Technology Co., Ltd., Jiangsu, China). To determine the O_2_^.-^ content, an O_2_^.-^ assay kit was used (Mengxi Biomedical Technology Co., Ltd., Jiangsu, China). Peroxidase (POD), catalase (CAT), superoxide dismutase (SOD), and malondialdehyde (MDA) detection kits (Mengxi Biomedical Technology Co., Ltd., Jiangsu, China) were used to determine the POD, CAT, and SOD activity and MDA content, respectively. Measurements were made according to the manufacturer’s instructions.

### 2.9 Determination of key enzymes and metabolites of the ascorbate–glutathione cycle

With and without LW-CNs treated wheat seeds under salt stress were used to determine key enzymes and metabolites in AsA-GSH cycle. Ascorbate peroxidase (APX), glutathione reductase (GR), monodehydroascorbate reductase (MDHAR), and dehydroascorbate reductase (DHAR) activities were determined using APX, GR, MDHAR, and DHAR assay kits (Mengxi Biomedical Technology Co., Ltd., Jiangsu, China), respectively, following the manufacturer’s instructions.

Dehydroascorbic acid (DHA) can be converted into reduced ascorbic acid, which can react with ferric ions (Fe^3+^) to form ferrous ions (Fe^2+^), reacting with red phenanthroline to form a red chelate with an absorption peak at 534 nm. The DHA content in the sample was determined by measuring the reduced ascorbic acid before and after the sample was reduced. To determine the oxidized glutathione (GSSG) content, 2-VP was combined with GSH to exclude GSH interference. GR catalyzed the redox reaction between GSSG and NADPH so that GSSG was reduced to GSH. GSH then reacted with 2-nitrobenzoic acid (DTNB) to form a chromogenic substance, which reacts to the amount of GSSG in the sample. To determine the GSH content, DTNB was reacted with GSH to form a complex, showing a characteristic absorption peak at 412 nm. Its absorbance was directly proportional to the GSH content. The reduced ascorbic acid content in wheat was determined using the AsA assay kit (Mengxi Biomedical Technology Co., Ltd., Jiangsu, China), following the manufacturer’s instructions.

### 2.10 Statistical analysis

All data were represented as mean ± SE and were analysed using SPSS 23.0. Comparisons were performed by either one-way ANOVA based on Duncan’s multiple range test (two tailed) or independent samples t-test (two tailed).

## 3 Results

### 3.1 Characterization of LW-CNs

**Figure 1:**
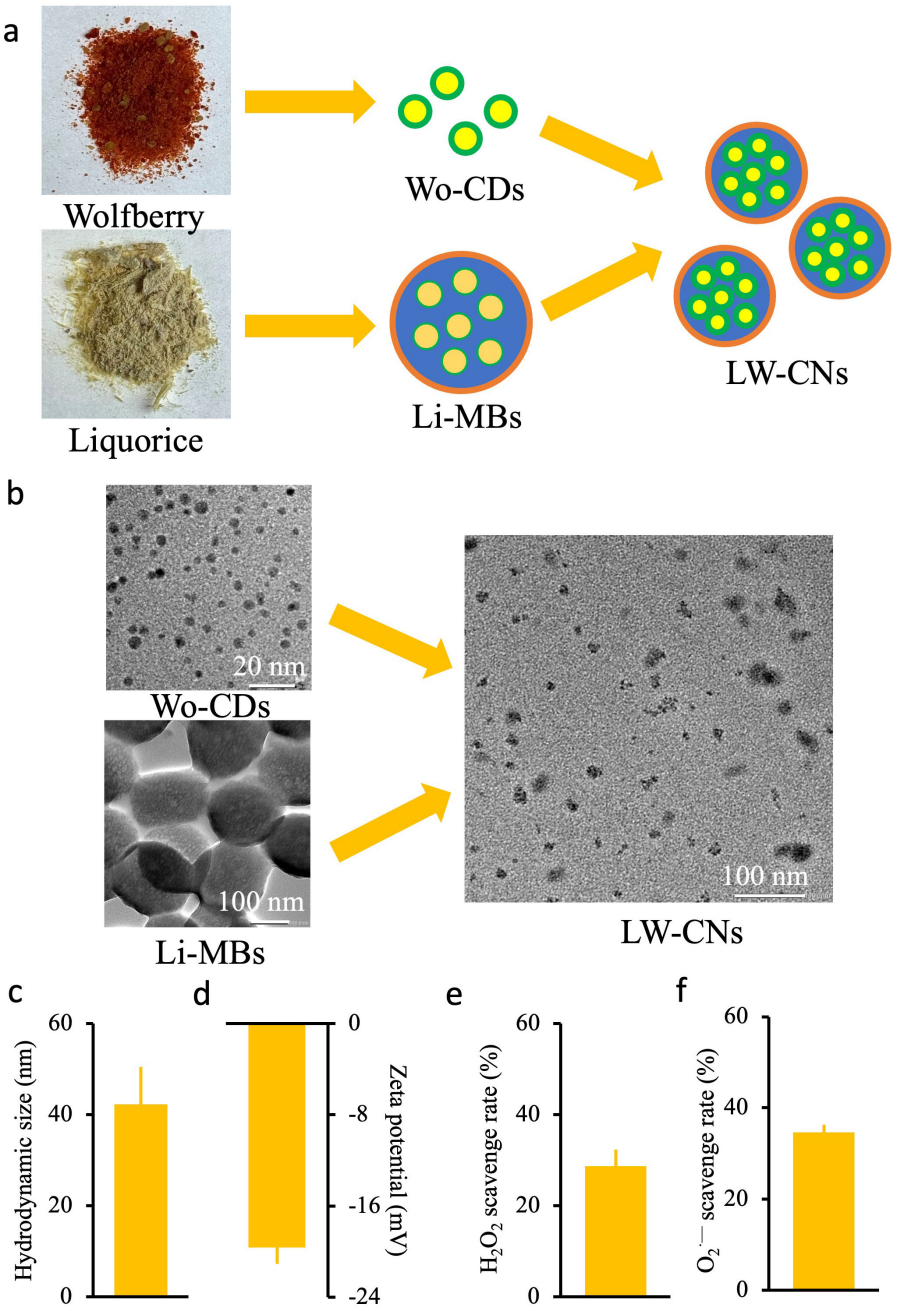
Synthesis and characterization of LW-CNs: **(a)** Schematic diagram of the synthesis of LW-CNs; **(b)** high resolution transmission electron microscopy (imaging of LW-CNs); **(c)** Hydrodynamic size of LW-CNs; **(d)** Zeta potential; **(e, f)** H_2_O_2_ and O_2_^·—^ scavenge rate. Error bar show Mean ± SE (*n* = 3).

Carbon-based nanomaterials (LW-CNs) with an average diameter of 13.87 ± 1.7 nm was prepared using licorice and wolfberry as raw materials and hydrothermal reactions at 160°C for 6 h (**Figure 1a**). The TEM image showed (**Figure 1b**) that Wo-CDs and Li-MSs were spherical, and the LW-CNs is a points doped sphere with smaller volumes than Li-MSs. Dynamic light scattering showed that the hydrodynamic dimensions of LW-CNs were 42.2 ± 8.2 nm (**Figure 1c**). The zeta potential measurement of LW-CNs showed that the surface charge was -19.6 ± 1.4 mV, and it had good particle dispersion (**Figure 1d**). We also measured the ROS clearance capacity of LW-CNs *in vitro*, and the H_2_O_2_ and O_2_^.-^clearance efficiencies of 200 mg L^-1^ LW-CNs were 28.7 and 34.6%, respectively (**Figures 1e, f**).

### 3.2 Influence of LW-CNs on wheat germination and phenotype

**Figure 2:**
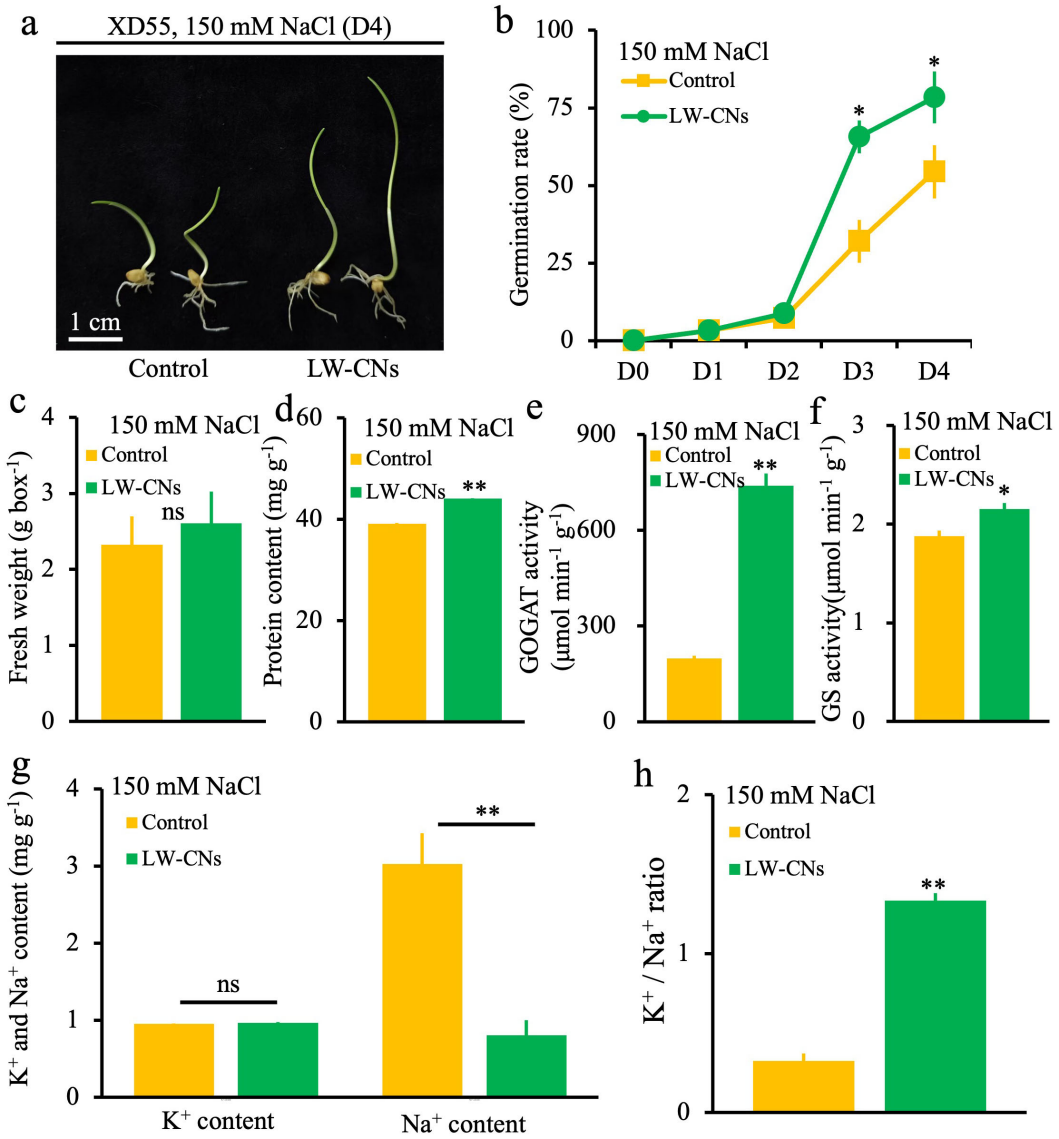
Phenotype of 4 days wheat seeds under salt stress with and without LW-CNs treatment. **(a)** Growth phenotype, scale bar = 1 cm; **(b)** germination rate; **(c)** fresh weight; **(d)** protein content; **(e)** GOGAT activity; **(f)** GS activity; **(g)** K⁺ and Na⁺ content ; **(h)** K⁺/Na⁺ ratio. A comparison between treatments was performed by independent sample t tests (two tailed) in **(b-f)**. ** indicate significance at p ≤ 0.01 levels, respectively. Mean ± SE (n = 3).

To determine the effects of LW-CNs on wheat’s germination ability, germination was compared between LW-CNs-treated and control wheat under 150 mM NaCl stress. As shown in **Figure 2a**, LW-CNs treatment resulted in a better growth phenotype than the control group. The germination rate was significantly higher under the LW-CNs treatment than under the control on the third day, increasing by 33.6%. On the fourth day, the germination rate increased by 24%, reaching 78.4% (**Figure 2b**). However, no significant difference in fresh weight was observed between the LW-CNs treatment group and the control group (**Figure 2c**). In contrast, the protein content in the LW-CNs-treated group increased significantly by 12% compared to the control group (**Figure 2d**). Without salt stress, after 4 days germination, there was no significant difference in the phenotype of wheat seeds between the LW-CNs treatment group and the control group. The germination rate reached 100% on Day 2, and no observed differences in fresh weight and MDA content (**Supplementary Figure S2**). As shown in **Figures 2e, f**, the GOGAT and GS in wheat treated with LW-CNs increased significantly by 271.6% and 14.7%, respectively. In comparison with the control group, the K^-1^ content of wheat seeds treated with LW-CNs under salt stress did not exhibit a significant difference, whereas the Na^-1^ content decreased significantly by 24% (**Figures 2g, f**). Under salt stress conditions, the ratio of potassium ions to sodium ions in wheat seeds treated with LW-CNs significantly increased by 75% (**Figure 2h**).

### 3.3 LW-CNs soaking increased the photosynthetic pigment content

**Figure 3:**
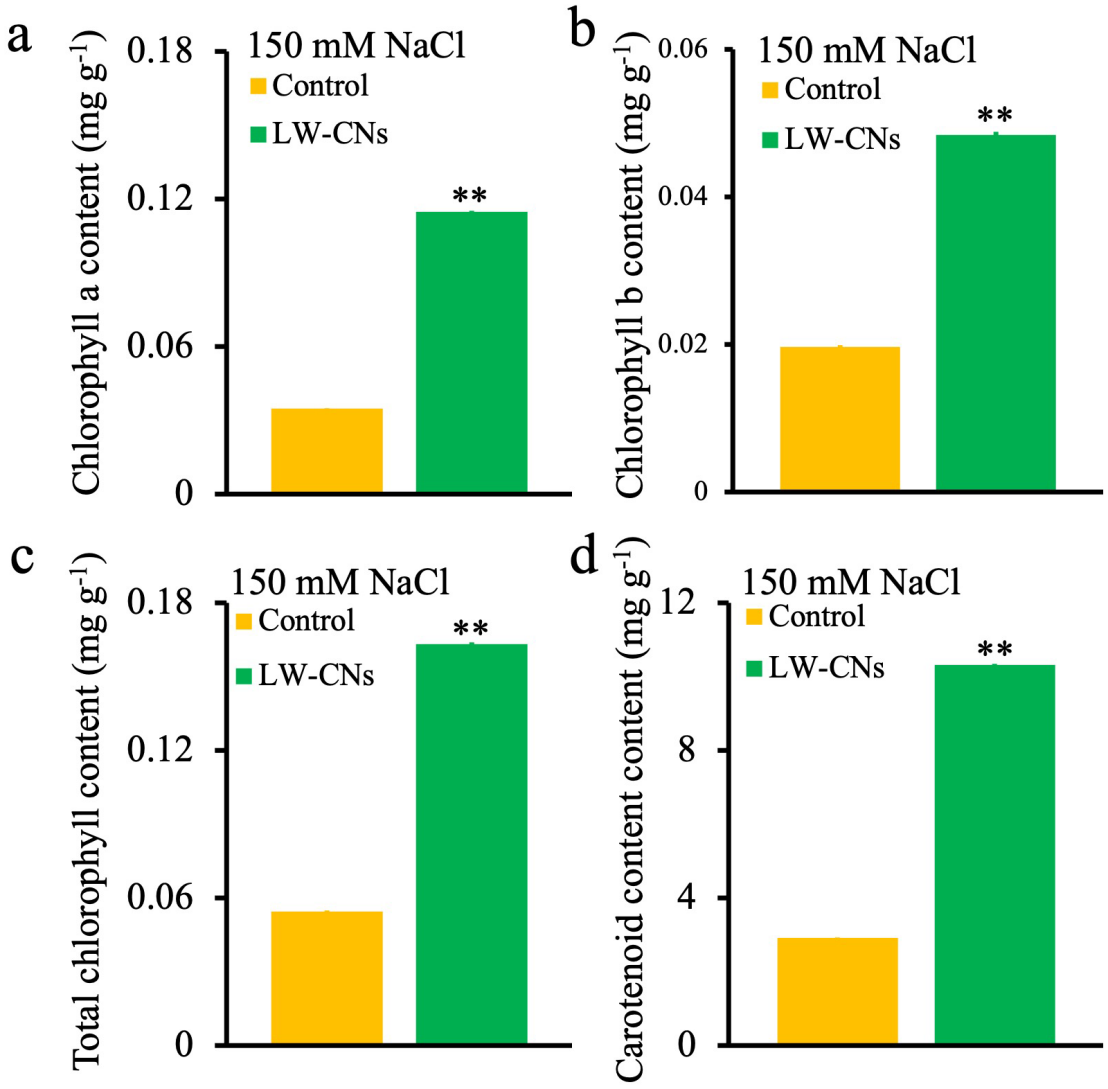
Chlorophyll content of 4 days wheat seeds under salt stress with and without LW-CNs treatment. **(a)** *Chlorophyll a* content; **(b)** *Chlorophyll b* content; **(c)** Total chlorophyll content; **(d)** Carotenoid content. A comparison between treatments was performed by independent sample t tests (two tailed) in (**a–d**). **indicate significance at *p<* 0.01 levels, respectively. Mean ± SE (*n* = 3).

Photosynthetic pigments are a class of pigment molecules in plants that are used to capture light energy and convert it into chemical energy, and they are the core components of photosynthesis. We measured the contents of photosynthetic pigments, including *chlorophyll a*, *chlorophyll b*, and carotenoids, in wheat under salt stress. As shown in **Figure 3**, the contents of *chlorophyll a*, *chlorophyll b*, total chlorophyll, and carotenoids in wheat under salt stress increased by 230, 146, 199, and 254%, respectively, in the LW-CNs treatment.

### 3.4 Effects of LW-CNs on antioxidant enzyme activity and ROS homeostasis

**Figure 4:**
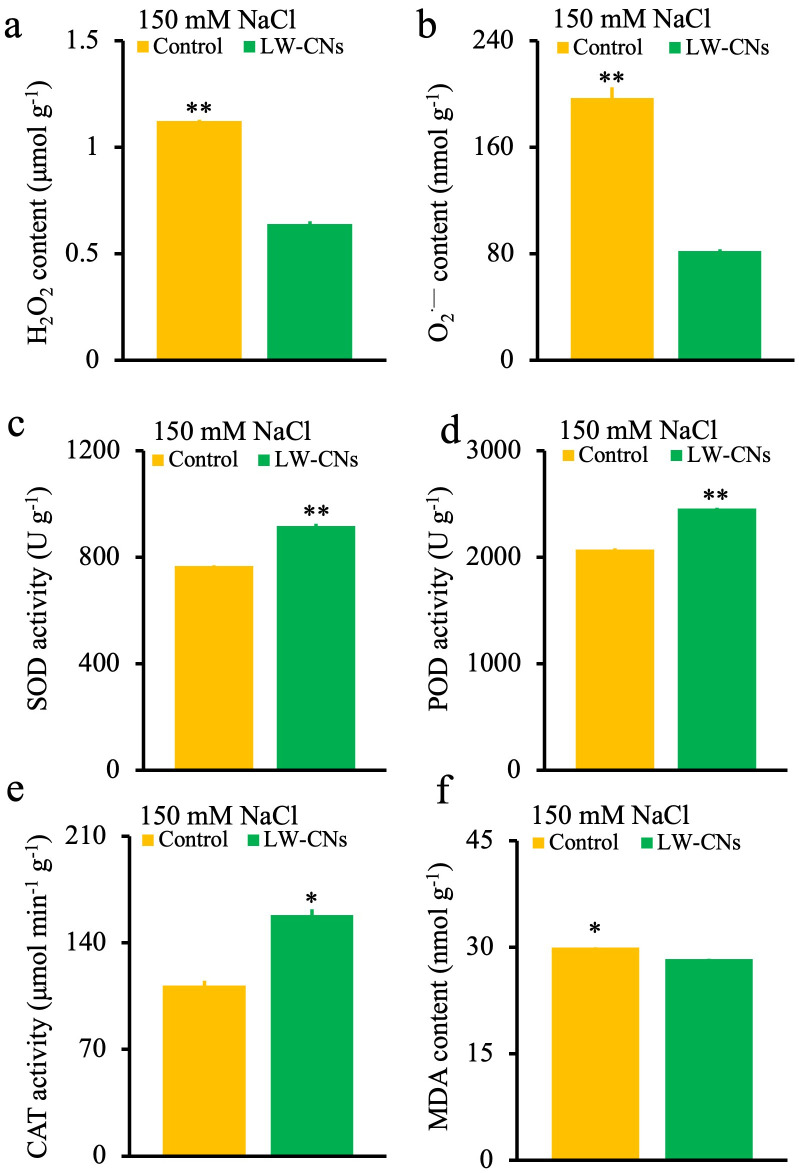
ROS content and antioxidase enzyme activity of 4 days wheat seeds under salt stress with and without LW-CNs treatment. **(a)** H_2_O_2_ content; **(b)** O_2_^·—^ content; **(c)** Superoxide dismutase (SOD) activity; **(d)** peroxidase (POD) activity; **(e)** catalase (CAT) activity; **(f)** malondialdehyde (MDA) content. A comparison between treatments was performed by independent sample t tests (two tailed) in **(a–f)**. * and ** indicate significance at *p*< 0.05 and 0.01 levels, respectively. mean ± SE (*n = 3*).

Under abiotic stress, the production of ROS, such as O_2_^.-^ and H_2_O_2_, causes oxidative stress, and the content of MDA, the final product of membrane lipid peroxidation, shows the degree of oxidative damage to plant tissues. In this study, as shown in **Figures 4a, b, f**, compared to the control group, the O_2_^·-^, H_2_O_2_, and MDA contents in wheat seeds treated with LW-CNs significantly decreased by 58, 43, and 5%, respectively. LW-CNs treatment significantly reduced the oxidative stress level in wheat under salt stress, indicating that the activity of antioxidant enzymes, as important components of the antioxidant system, was regulated by LW-CNs. As shown in **Figures 4c–e**, the SOD, POD, and CAT activities significantly increased by 19.5, 18.7, and 41.3%, respectively, under LW-CNs treatment. LW-CNs treatment enhanced the enzyme activity. These findings suggest that LW-CNs alleviates excess ROS accumulation in plants by activating antioxidant enzymes, such as SOD and CAT, thereby enhancing salt tolerance in wheat.

### 3.5 Effect of LW-CNs on the AsA-GSH cycle in wheat under salt stress

**Figure 5:**
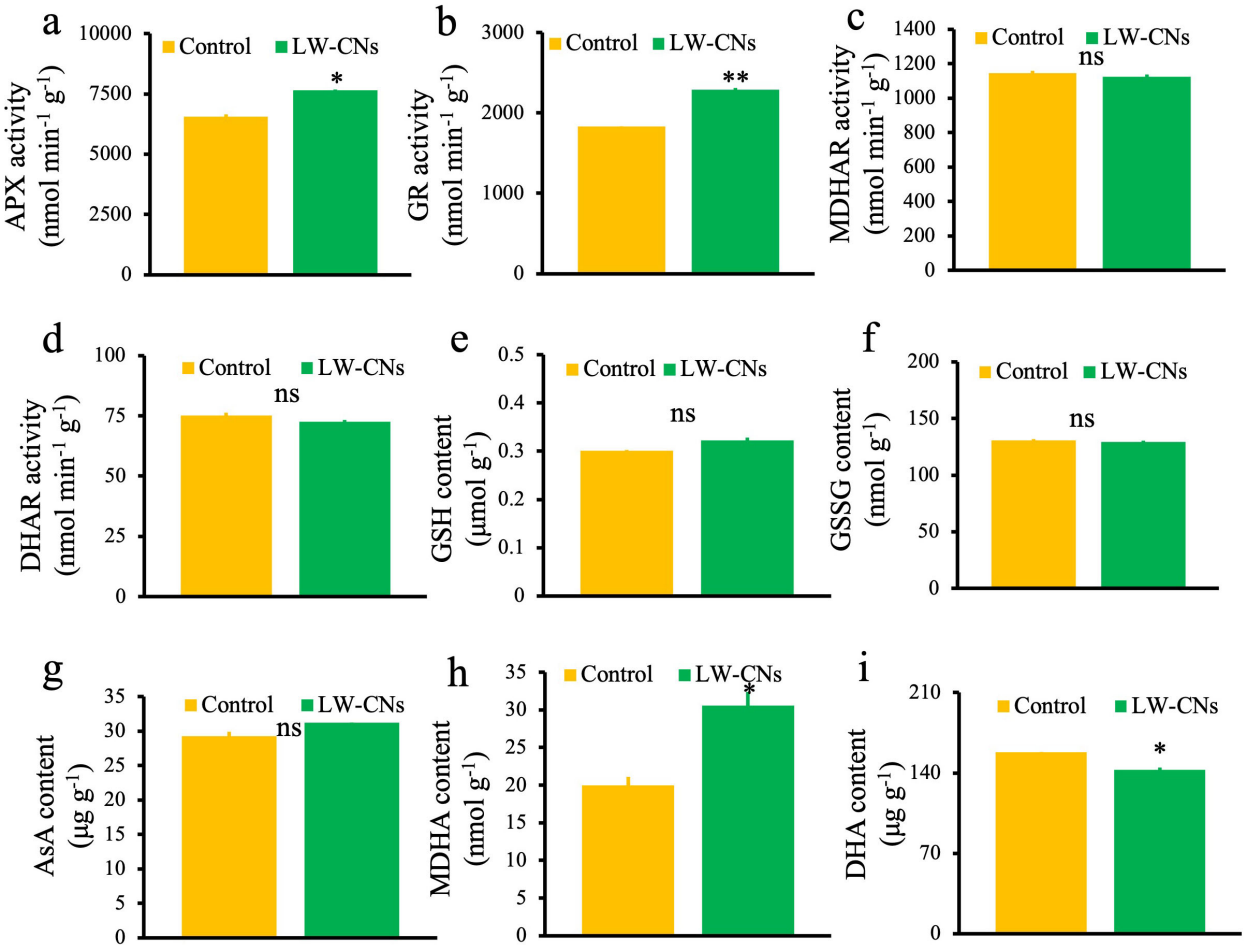
The oxidation Key enzymes activity and metabolites content in the AsA-GSH cycle of 4 days wheat seeds under salt stress with and without LW-CNs treatment. **(a)** Ascorbate peroxidase (APX) activity; **(b)** gluathione reductase (GR) activity; **(c)** monodehydroascorbate reductase (MDHAR) activity; **(d)** dehydroascorbate reductase (DHAR) activity; **(e)** glutathione (GSH) content; **(f)** glutathione, oxidized (GSSG) content; **(g)** ascorbic acid (AsA) content; **(h)** Monodehydroascorbate reductase (MDHA) activity **(i)** Hexaenoie Acid (DHA) content. A comparison between treatments was performed by independent sample t tests in **(a–i)**. * and ** indicate significance at *p*< 0.05 and 0.01 levels, respectively. Mean ± SE (*n = 3*), ns indicates no significant difference.

We measured the key enzymes activity and metabolites content in the AsA-GSH cycle. Among these, the activities of APX (which scavenges H_2_O_2_) and GR (which generates crucial antioxidants) significantly increased by 16.5 and 24.9%, respectively (**Figures 5a, b**). In contrast, the MDHAR and DHAR activities showed no significant differences (**Figures 5c, d**). GSH and AsA, as critical antioxidants in the AsA-GSH cycle, exhibited no significant differences between the LW-CNs treatment and control groups (**Figures 5e, g**). Additionally, under LW-CNs treatment, the content of GSSG (the oxidized form of GSH) increased by 0.7% compared to the control, although not significantly (**Figure 5f**). The MDHA content showed a significant increase of 52.7% compared to the control (**Figure 5h**). DHA is generated through the spontaneous disproportionation reaction of MDHA, and the DHA content was significantly reduced by 9.7% under LW-CNs treatment (**Figure 5i**).

### 3.6 Effect of LW-CNs on the antioxidant defense network and ROS metabolism in salt-stressed wheat revealed by correlation and PCA

**Figure 6:**
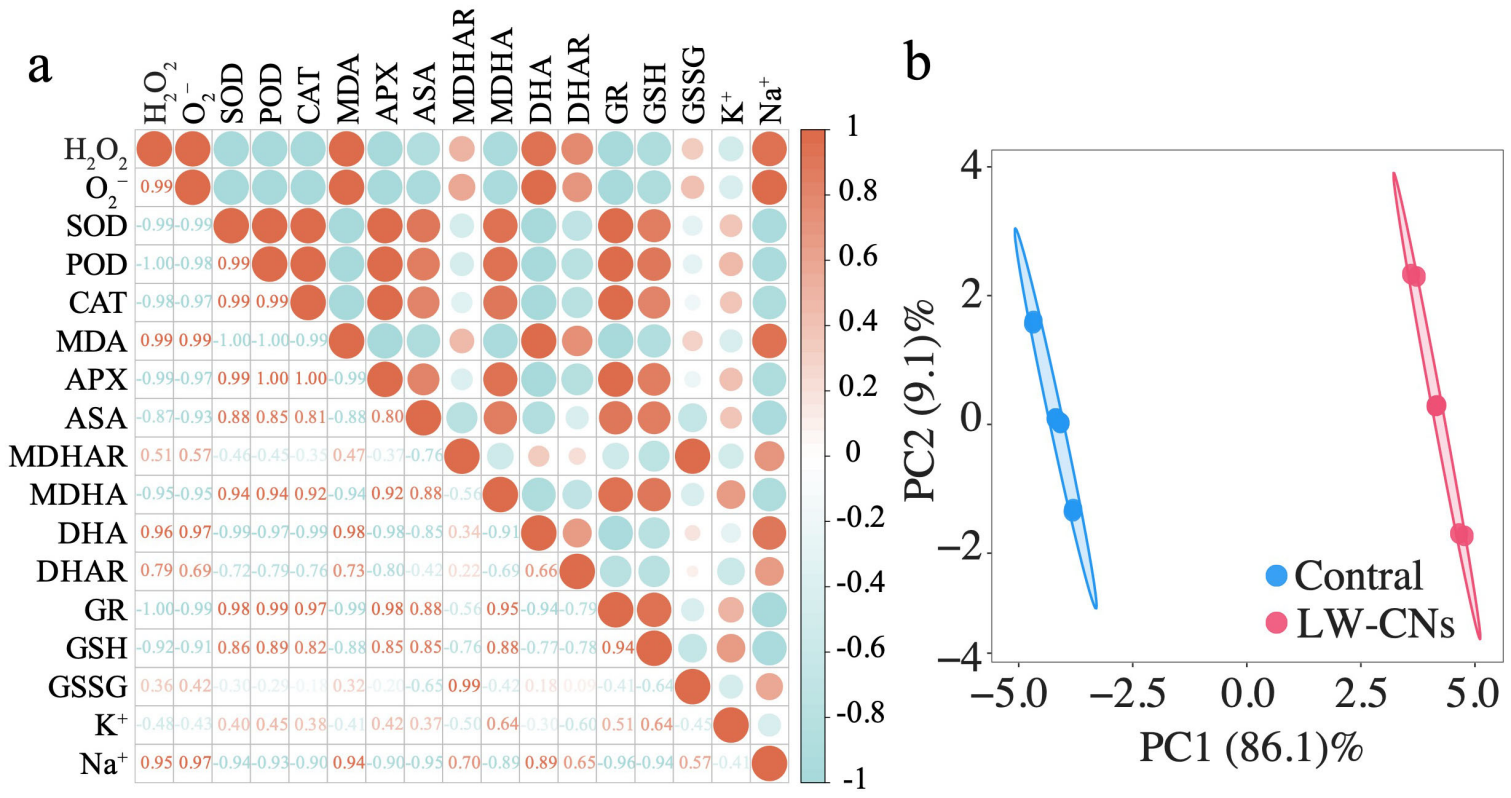
Effect of LW-CNs on the antioxidant defense network and ROS metabolism in salt-stressed wheat revealed by correlation and PCA. **(a)** Correlation analysis between antioxidant indices, ROS, MDA markers, and K^-1^/Na^-1^ content. **(b)** Principal component analysis (PCA) of oxidative stress parameters.

As shown in **Figure 6a**, the correlation analysis demonstrates the relationships between reactive oxygen species (ROS) metabolism indices and K^-1^/Na^-1^ content in wheat under salt stress. Antioxidant indices (SOD, POD, CAT, APX, AsA, GR) negatively correlate with ROS and MDA markers (•O_2_^-^, H_2_O_2_, MDA). In the AsA-GSH cycle, MDHAR and DHAR convert MDHA and DHA to AsA, while GR reduces GSSG to GSH. Notably, MDHAR and DHAR show significant positive correlations with ROS and MDA, whereas MDHA exhibits negative correlations with these oxidative markers. GR and GSH negatively correlate with GSSG but positively correlate with SOD, POD, and CAT. K^-1^ content negatively relates to antioxidant, ROS, and MDA indices, in contrast to Na^-1^. Overall, antioxidant indices positively correlate with ROS, MDA within groups but negatively correlate between groups. Principal component analysis (PCA) was conducted after data standardization (mean=0, SD=1). The first two principal components (PC1 and PC2) had eigenvalues >1 and cumulatively explained 95.8% of total variance, with PC1 alone accounting for 86.1%. PC1’s high loadings (>0.7) include H_2_O_2_ and •O_2_⁻ (Loading=-0.997), as well as GR (0.998), SOD (0.989), and POD (0.991), indicating that PC1 represents redox balance: positive loadings link to antioxidant enzymes (GR, SOD, POD), while negative loadings correspond to ROS accumulation (H_2_O_2_, •O_2_⁻) (**Supplementary Figure S2**). **Figure 6b** reveals complete separation between Control and LW-CNs on PC1: LW-CNs cluster in the positive region (mean=4.05 ± 0.35), while Control distributes in the negative region (mean=-3.98 ± 0.33), demonstrating that LW-CNs enhance antioxidant defense and suppress ROS.

## 4 Discussion

### 4.1 LW-CNs enhances wheat development under salt stress

The ingress of nanomaterials into seeds is a continuous process, which may differ due to the thickness of the seed coat. Previous studies have revealed that when rapeseed seeds were treated with nanomaterials, the entry of nanomaterials into the seed tissue could be distinctly observed at approximately 3 hours, and obvious nanomaterial signals could be detected in the cotyledons and radicles of the seeds after 8 hours. In contrast to rapeseed seeds, the seed coat of wheat seeds is thinner, and excessive entry of nanomaterials might lead to toxic effects. Hence, we selected 3 hours as the treatment duration for LW-CNs on seeds. Normal nanomaterials that facilitate crop growth under salt stress are mostly composed of a single morphology. Currently, most studies evaluate nanoparticles or carbon dots. As delivery systems, nanomaterials, such as mesoporous silica (MSNs), enhance or protect the efficacy of the loaded reagents (Niu et al., 2024). In our study, Wo-CDs, a type of carbon dot, were formed by a single component. Li-MBs are mesoporous spheres with a relatively large particle size and a structure similar to that of MSNs. When the two raw materials react together, Wo-CDs are loaded onto Li-MBs to form LW-CNs. Compared to Li-MBs, the volume of LW-CNs decreased, which might be more beneficial for LW-CNs to enter plant cells (Qiu et al., 2025).

We employed the seed soaking method to apply LW-CNs to wheat seeds. After 4 days of salt stress, LW-CNs facilitated the growth and germination of wheat seeds, but the fresh weight did not increase. This was because photosynthesis did not occur during the early stage of seed germination. Our experiment was carried out under weak light; thus, plant did not effectively accumulate assimilates. However, the increased protein content in LW-CNs-treated wheat seeds was comprehensible due to their intensified physiological activities compared to the control group, where salt stress severely hindered growth. This was specifically demonstrated by LW-CNs treatment increasing GOGAT and GS activities by 271.6% and 14.7% respectively (**Figures 2e, f**), consistent with elevated protein content, while concurrently reducing Na^-1^ content by 24% (**Figure 2g**) and maintaining K^-1^ levels, indicating effective alleviation of ionic imbalance under salt stress. The increase in the photosynthetic pigment contents also indicates that LW-CNs promoted wheat seed growth under salt stress.

### 4.2 LW-CNs maintains ROS homeostasis to protect wheat seeds from salt stress

ROS represent a double-edged sword within plant cells. Conventionally, ROS serve as a signaling molecule, functioning as a secondary messenger in the development of plants and their responses to external stress. Nevertheless, in the context of salt stress, the loss of water and ion imbalance result in substantial ROS accumulation, thereby resulting in proteins, lipids, pigments, and DNA damage and subsequently influencing plant growth and development (He et al., 2024; Kruk et al., 2005; Li et al., 2022; Wang et al., 2008).

The results of this research demonstrate that LW-CNs have good scavenging abilities for H_2_O_2_ and O_2_^·—^, which is in line with the ROS levels in wheat seeds after soaking treatment. This indicates that LW-CNs enables the wheat seeds to eliminate excessive ROS accumulation under salt stress and reduce MDA generation resulting from plasma membrane damage. Our results further demonstrate that maintaining ROS homeostasis using nanomaterials is an important approach for enhancing the salt tolerance of crops.

To date, the mechanisms for maintaining the ROS homeostasis of plants and enhancing plant salt tolerance through nanobiotechnology mainly include the following: 1) nanomaterials with ROS scavenging capabilities acting as antioxidant enzymes to eliminate excessive ROS accumulation (Liu et al., 2023; Qiu et al., 2025); 2) nanomaterials increasing the activity of antioxidant enzymes in crops and lowering ROS levels (Li et al., 2022; Liu et al., 2023, 2021b; Lu et al., 2020; Qiu et al., 2025; Zhou et al., 2021); and 3) nanomaterials improving the salt tolerance of crops by stimulating ROS generation and stress training the plants (Chen et al., 2023). In the present study, soaking wheat seeds with LW-CNs effectively increased the SOD, POD, and CAT activities of wheat seeds under salt stress, further maintaining ROS homeostasis. Khan et al (Khan et al., 2021). utilized cerium oxide nanoparticles and Se-CDs to enhance the salt tolerance of rape, and the principal mechanism was maintaining the ROS balance.

### 4.3 The AsA-GSH cycle plays a significant role in LW-CNs, improving wheat’s salt tolerance

The hydroxyl radical is regarded as one of the most destructive ROS, and currently, no enzymatic system has been reported to eliminate hydroxyl radicals. AsA can eliminate hydroxyl radicals, mainly by providing electrons to the hydroxyl radicals to convert them into harmless water. After reacting with hydroxyl radicals, AsA transforms into DHA, which is then reduced by DHAR (Bao et al., 2024). In our study, both the DHA content and DHAR activity decreased, indicating that wheat seeds soaked in LW-CNs did not clear hydroxyl radicals through AsA. GSH also directly neutralizes hydroxyl radicals through its sulfhydryl groups, and GSH can be reduced by GR. In our study, the GR activity increased, but the GSH content did not, indicating that the main method for LW-CNs-treated wheat seeds to eliminate hydroxyl radicals is through the neutralization of hydroxyl radicals by GSH.

**Figure 7:**
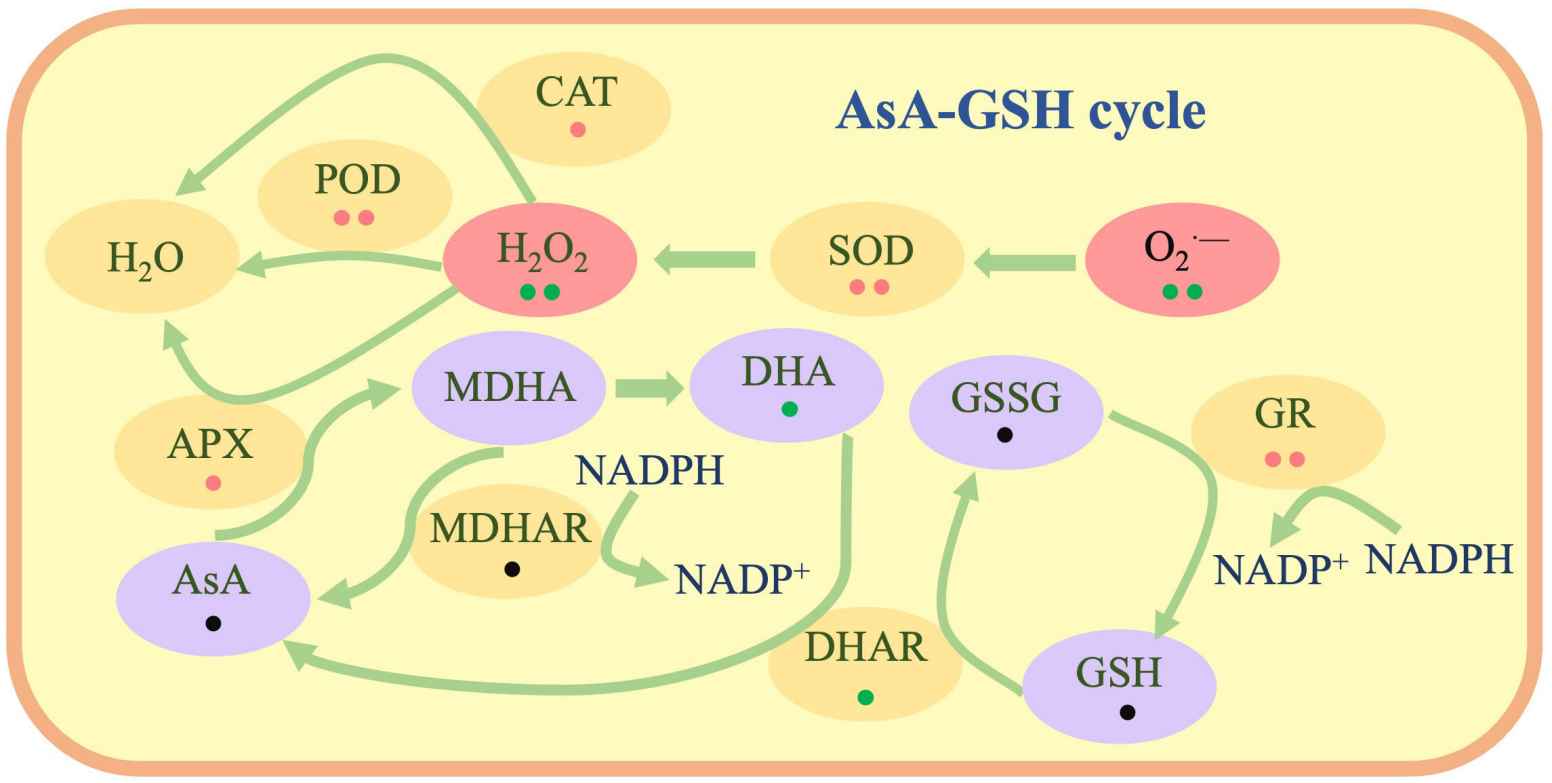
The schematic diagram illustrates the mechanism by which LW-CNs soaking treatment effectively maintains reactive oxygen species (ROS) homeostasis in wheat plants through two synergistic pathways: enhancing the functional capacity of antioxidant enzymes and preserving the integrity of the ascorbate-glutathione (AsA-GSH) metabolic cycle. Red points indicate significant increase in LW-CNs treated seeds, green points indicate no significant between LW-CNs treated seeds and control, black points indicate decrease in LW-CNs treated seeds. One point shows *p<* 0.05, two points shows *p<* 0.01.

In the AsA-GSH cycle, similar to POD and CAT, APX oxidizes AsA to MDHA and eliminates hydrogen peroxide, and this process is associated with the SOD process of eliminating. MDHA can be reduced to AsA by MDHAR (Liu et al., 2021a). We evaluated the substance content and enzyme activity related to the AsA and GSH cycles in wheat seeds under salt stress. Soaking seeds with LW-CNs had no significant effect on the AsA and GSH contents, but the content of their upstream substrates (MDHA) and enzyme activity (GR) increased. This might be because AsA and GSH are involved in ROS scavenging. The decrease in the DHA content in wheat seeds soaked in LW-CNs increased the AsA/DHA ratio, which is regarded as a sign of plant stress tolerance enhancement (**Figure 7**).

### 4.4 Natural nanomaterials are of great significance for food yield increases

Under With the increase in the population, the demand for food is growing day by day. Nanobiotechnology is a new technology for increasing crop yield. Nanobiotechnology has agricultural applications, such as for enhancing stress resistance and delivering pesticides, nano-fertilizers, and nano-sensors (Bao et al., 2024; Chhipa, 2017; Giraldo et al., 2019; Liu et al., 2021b). Among these agricultural nanomaterials, those synthesized from industrial raw materials are predominant. However, this increases the cost of agricultural production and poses risks to biological safety. Using herb synthesis of nano-materials might be a better option. For instance, in our research, licorice and goji berries were used, with a price of approximately 0.5 RMB/g. This significantly reduces the cost of raw materials for agricultural nano-materials. Li et al. found that spraying carbon dots derived from *Salvia miltiorrhiza* on leaves enhanced the salt tolerance of crops (Li et al., 2021). Seed soaking might be a more economical method, as it requires less product. Therefore, the use of nano-materials derived from herbs can effectively enhance crop yields.

## 5 Conclusions

Salt stress limits plant growth. Nano-soaking, which has environmental and economic advantages, is an emerging method for enhancing plant salt tolerance during the germination period. However, knowledge about the mechanisms behind the improvement of salt tolerance through nano-soaking during the germination period is limited. To the best of our knowledge, the role, and underlying mechanisms of LW-CNs in improving plant salt stress tolerance have hardly been reported. LW-CNs treatment rapidly enhanced wheat germination under salt stress by maintaining ROS homeostasis and promoting nitrogen metabolism. Subsequently, the AsA-GSH cycle was evaluated to investigate how LW-CNs maintained wheat ROS homeostasis under stress. GSH mainly participates in the scavenging of hydroxyl radicals, while AsA mainly clears hydrogen peroxide. Overall, LW-CNs maintained the ROS homeostasis in wheat seeds under salt stress by enhancing the activity of antioxidant enzymes and increasing the contents of GSH and AsA, thereby enhancing the germination rate. Furthermore, the measurement of the K^-1^/Na^-1^ ratio in plants under salt stress remains a valid approach for assessing the biological effects of plant - derived nanomaterials. A high K^-1^/Na^-1^ ratio serves as an indicator of the high salt tolerance capacity in crops. The findings of our study provide more insight into the mechanism by which herbal-derived nanomaterials induce plant salt tolerance during the germination period.

## Data Availability

The original contributions presented in the study are included in the article/**Supplementary Material**. Further inquiries can be directed to the corresponding authors.

## Supplementary material

Supplementary Figure 1
**(a)** Autoclave reactor used for hydrothermal synthesis of LW-CNs; **(b)** Germination device; **(c)** Constant-temperature illuminated incubator.

Supplementary Figure 2 Loadings plot of PCA.
