# Supplementary figures and images for: Licorice–wolfberry-derived nanomaterial improves the germination rate of wheat under salt stress by maintaining reactive oxygen species homeostasis
Source: Front Plant Sci. 2025 Sep 8;16:1657516. doi: 10.3389/fpls.2025.1657516 (PMC12450925; doi:10.3389/fpls.2025.1657516)

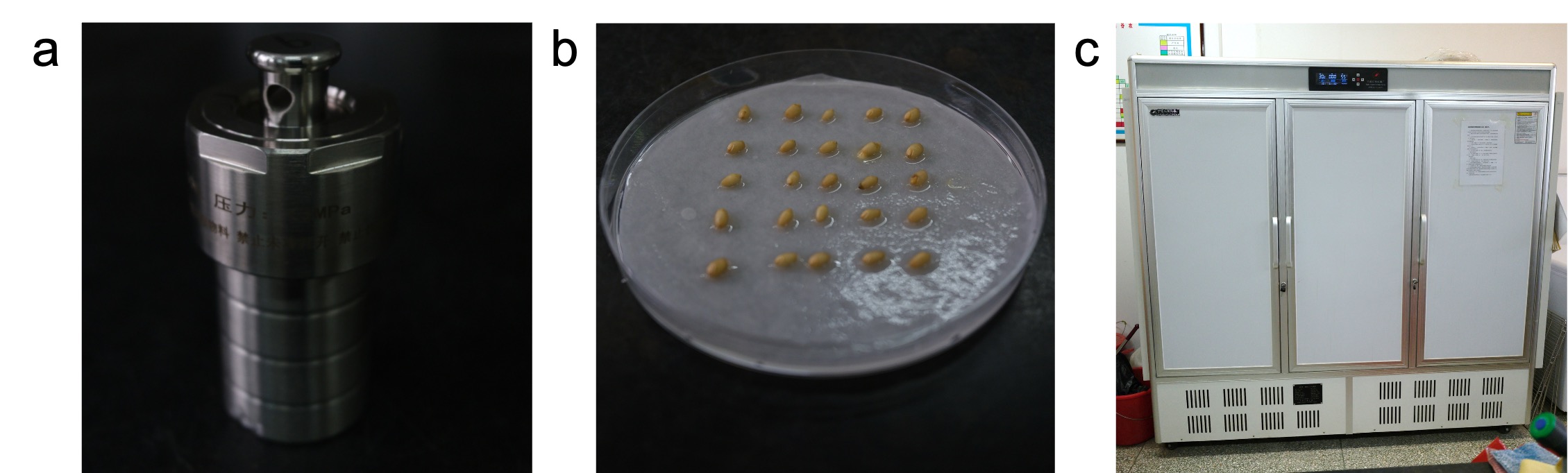

Supplement: Supplementary Figure 1 — (a) Autoclave reactor used for hydrothermal synthesis of LW-CNs; (b) Germination device; (c) Constant-temperature illuminated incubator. [file Image1.jpeg]

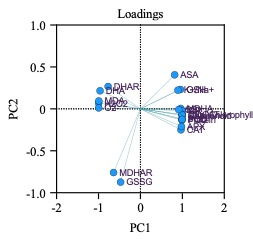

Supplement: Supplementary Figure 2 — Loadings plot of PCA. [file Image2.jpeg]
